# Prospective Evaluation of Whole Genome MicroRNA Expression Profiling in Childhood Acute Lymphoblastic Leukemia

**DOI:** 10.1155/2014/967585

**Published:** 2014-05-14

**Authors:** Muhterem Duyu, Burak Durmaz, Cumhur Gunduz, Canan Vergin, Deniz Yilmaz Karapinar, Serap Aksoylar, Kaan Kavakli, Nazan Cetingul, Gulersu Irken, Yontem Yaman, Ferda Ozkinay, Ozgur Cogulu

**Affiliations:** ^1^Department of Pediatrics, Faculty of Medicine, Ege University, 35100 Izmir, Turkey; ^2^Department of Medical Genetics, Faculty of Medicine, Ege University, Bornova, 35100 Izmir, Turkey; ^3^Department of Medical Biology, Faculty of Medicine, Ege University, 35100 Izmir, Turkey; ^4^Department of Pediatric Hematology, Behcet Uz Children's Hospital, 35210 Izmir, Turkey; ^5^Department of Pediatrics, Faculty of Medicine, Dokuz Eylul University, 35340 Izmir, Turkey

## Abstract

Dysregulation of microRNA (miRNA) expression contributes to the pathogenesis of several clinical conditions. The aim of this study is to evaluate the associations between miRNAs and childhood acute lymphoblastic leukemia (ALL) to discover their role in the course of the disease. Forty-three children with ALL and 14 age-matched healthy controls were included in the study. MicroRNA microarray expression profiling was used for peripheral blood and bone marrow samples. Aberrant miRNA expressions associated with the diagnosis and outcome were prospectively evaluated. Confirmation analysis was performed by real time RT-PCR. miR-128, miR-146a, miR-155, miR-181a, and miR-195 were significantly dysregulated in ALL patients at day 0. Following a six-month treatment period, the change in miRNA levels was determined by real time RT-PCR and expression of miR-146a, miR-155, miR-181a, and miR-195 significantly decreased. To conclude, these miRNAs not only may be used as biomarkers in diagnosis of ALL and monitoring the disease but also provide new insights into the potential roles of them in leukemogenesis.

## 1. Introduction

The prevalence of cancer is 11–15/100.000 among children and leukemia is the most common malignancy with an incidence of 30.2%, hence a major cause of mortality and morbidity [1, 2]. Acute lymphoblastic leukemia (ALL) accounts for 75% of childhood leukemia [2]. Event-free-survival (EFS) rates of childhood ALL exceeded 80% with current treatment protocols and relapse as the main reason for therapy failure [3]. Pediatric leukemia has distinct features when compared to adult leukemia, especially in prognosis and treatment options [4]. There are also individual differences among patients of the same age and risk group in response to treatment and in prognosis. Genetic factors are seen as one of the likely culprits for these discrepancies. Recently, microRNAs have been shown to be important molecules in developmental and oncogenic processes. They are 19–25 nt long posttranscriptional regulator ribonucleic acid (RNA) molecules [5]. They target mRNAs, inhibit the translation into proteins, and silence specific gene expression [6]. Aberrant expression of miRNAs has been observed in many types of cancer, showing either tumor suppressive or oncogenic activity [7, 8]. The number of studies regarding the relationship between adult cancers and miRNAs is gradually increasing; however, there are still a relatively low number of studies regarding childhood malignancies. Moreover, there have been even fewer studies focusing specifically on the association of miRNAs with childhood ALL [9–15]. The aim of the present study is to identify the relevant miRNAs in the diagnosis of childhood ALL and evaluate their effect in the course of the disease.

## 2. Materials and Methods

### 2.1. Patients

A total of 45 children with newly diagnosed and untreated childhood ALL and 15 age-matched control subjects (normal peripheral blood (PB) and bone marrow (BM) smears) were enrolled in the study. Patients were consecutively included from the inpatient oncology and hematology departments of 3 hospitals. Previously diagnosed leukemia cases were excluded from the study as the miRNA expression levels might have been altered as a result of previous treatment. ALL diagnoses were confirmed via a BM aspirate showing at least 30% blast cells, in accordance with the FAB classification. All patients were diagnosed according to standard morphological, cytochemical, and immunophenotypic criteria. Patients were treated primarily with Berlin-Frankfurt-Munster- (BFM-) based national ALL protocol. This protocol was modified slightly with regard to methotrexate dosing and cranial irradiation. Following the BFM-ALL 1995 protocol, risk groups were categorized as standard (SRG), intermediate (IRG), and high (HRG) risk groups based on their age, leukocyte count, immunophenotyping, cytogenetic changes, early response to prednisone therapy, and BM remission. Patients received four drug induction regimens consisting of prednisone, asparaginase, vincristine, and daunorubicin. Complete remission (CR) was defined as a normocellular marrow with less than 5% blast cells. Patients' characteristics, such as age, sex, white blood cell (WBC) count, FAB classification, and treatment response, are available in Table 1.

### 2.2. Study Design

The cases were evaluated prospectively. The enrollment period for each patient in the study was 18 months, plus 2 years of followup. MicroRNA microarray profiling was performed on all patients at day 0 and in control cases. The significant miRNAs were confirmed by real time RT-PCR. Six months after treatment, significantly dysregulated and validated miRNAs at day 0 were again analyzed by real time RT-PCR. The change in miRNA levels over time was determined by comparing the ratio at day 0 with the ratio of those measured at 6 months. During this period, 4 patients died. Among our study population, 3 cases (2 from patient group and 1 from control group) were also excluded following RNA isolation process, due to insufficient signalization during microarray work. The significance of miRNAs that were obtained from PB samples was compared with BM samples. Aberrant miRNA expressions associated with the diagnosis, differential diagnosis, and outcome of ALL were evaluated.

### 2.3. RNA Extraction and Microarray Study

RNA isolation was performed on each BM and peripheral blood (PB) samples obtained from both patient and control groups. Total RNA was isolated by using Qiazol, which was then followed by miRNeasy Mini Kit (Qiagen, Valencia, USA) as per the manufacturer's instructions. Genome wide microRNA microarray profiling was performed by using a microRNA biochip platform (Febit, Heidelberg, Germany). The platform consisted of 1136 microRNA probes (Sanger, miRBase 12.0). In short, 0.7 micrograms of microRNA was labelled using miRVANA labeling kit (Ambion, USA) and then dried via SpeedVac (Thermo, Germany). Dried samples were then treated with 18 microL of hybridization buffer (Febit Biomed GmbH, Heidelberg, Germany) and placed into the biochip platform overnight. Following hybridization and washing, signals were measured. The signal enhancement procedure was processed with Geniom Real Time Analyzer (GRTA) and detection pictures were evaluated by using Geniom Wizard software. The signal intensities for all miRNAs were extracted from the raw data for each array. After background correction, the median signal intensity of the seven replicate intensity values of each miRNA was obtained. Normalization was conducted by using the freely available R software (http://www.R-project.org/).

The RNA quality control was determined by using the NanoDrop ND-1000 in which the ratios of 230/260 and 260/280 were 2. The RNA integrity number was determined by using Agilent 2100 Bioanalyzer (Agilent Technologies) and was ≥7.

The array data including raw data has been deposited in Gene Expression Omnibus (GEO) with the accession number GSE56489.

### 2.4. Quantitative Real Time Polymerase Chain Reaction and Validation

Significantly dysregulated miRNAs were validated by Quantitative PCR (LightCycler 480, Roche Applied Science, Mannheim, Germany) after comparing the BM microarray miRNA expressions of patients with the controls. RNA was reverse-transcribed to cDNA by using a cDNA synthesis kit (Exiqon). For miRNA quantification, the miRCURY LNA Universal RT microRNA PCR system (Exiqon) was used in combination with the predesigned primers (Exiqon). A master mix was designed for each primer set in accordance with the recommendations of the real time RT-PCR setup for “individual assays,” suggested in the kit. The reaction conditions consisted of polymerase activation/denaturation at 95°C for 10 min. For miRNA quantification, 40 amplification cycles at 95°C for 10 sec and 60°C for 1 min were performed; this was then followed by signal detection. The Delta-Delta-Ct algorithm was used to determine relative gene expression, and SNORD48 and U6 were used for housekeeping genes.

### 2.5. Statistical Analysis

According to mean values, only those miRNAs whose “fold change” value demonstrated ±2-fold or more expression difference were included in the study. miRNAs whose “False Discovery Rate” (FDR) corrected *P* value <0.05 were considered significant. FDR was determined by CLC Main Workbench 5 (CLC Bio, Denmark). Heat map and cluster analysis were performed for grouping of the miRNA data. Student's* t*-test was used to make comparisons in different groups by CLC Main Workbench.

### 2.6. Consent

This study was approved by the Local Ethics Committee, and written consent was taken from all parents of children that participated in the study.

## 3. Results

The patient group consisted of 43 cases with ALL in which 34 cases (79%) were B-lineage, and 9 cases (21%) were T-lineage ALL. The mean age in the ALL group was 6.8 ± 4.5. Fourteen cases constituted the control group and the mean age was 6.6 ± 5.1. The ratio of female/male was calculated 0.9 in ALL and 0.8 in the control group (Table 1). The distribution of ALL cases, according to the risk groups, was as follows: 13 cases (30%) were in the SRG, 20 cases (47%) were in the IRG, and 10 cases (23%) were in the HRG. Upon followup, 4 ALL patients (9%) (2 cases with SRG B-lineage ALL, 2 cases with HRG B-lineage ALL) died due to infectious complications (Table 1). Risk groups and response to the treatment of ALL cases are summarized in Table 1.

### 3.1. miRNA Analysis

**Figure 1:**
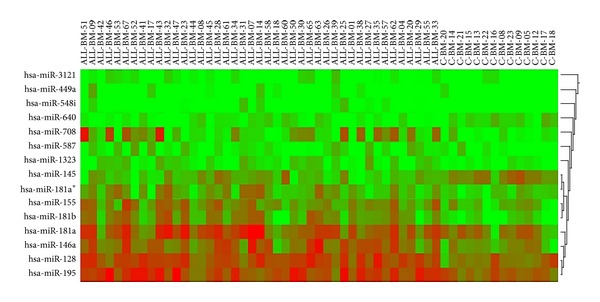
Heat map and cluster analysis in ALL patients and control cases (FDR *P* value <0.05). The figure shows the relative expression of bone marrow (ALL-BM) miRNAs in ALL patients and control cases (c-BM). miRNAs are in columns representing the 15 miRNAs, samples in rows representing 43 ALL patients and 14 control cases. The color scale shown on the top illustrates the expression level of the indicated miRNA across all samples: red means that a miRNA expression value is higher than its average expression across all samples (upregulated), and green means a lower expression value (downregulated).

**Figure 2:**
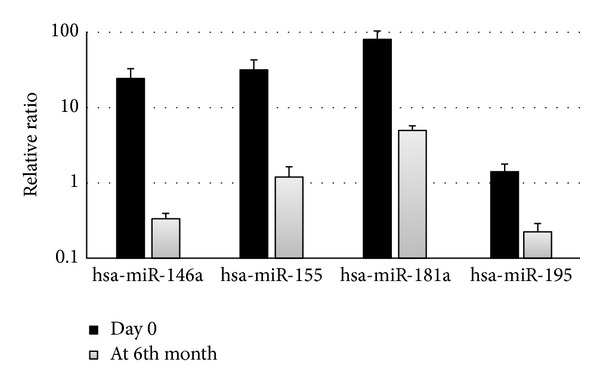
Diagram of significantly changed miRNAs in ALL patients after 6 months of treatment. The figure shows the change in expression levels of 4 significantly dysregulated miRNAs after 6 months of treatment. The change in miRNA levels over time was determined by comparing the ratio at day 0 with the ratio of those measured at 6 months by using quantitative real time RT-PCR.

miRNAs from PB samples were not correlated with BM samples. Significant miRNAs in PB of patients when compared to control cases were totally different to miRNAs in BM. Therefore, all analysis and considerations in different categories were made by using BM samples. We propose using BM samples for the miRNA analysis in hematological malignancies because they reflect the leukemic process more efficiently, when compared to PB. With reference to the controls' (*n* = 14), significantly dysregulated miRNAs in BM samples according to the microarray study (*n* = 43) are shown in Table 2. A total of 13 miRNAs (miR-548i, miR-708, miR-181b, miR-449a, miR-146a, miR-155, miR-181a, miR-3121, miR-181a, miR-128, miR-1323, miR-195, and miR-587) showed upregulation and 2 miRNAs (miR-640, miR-145) showed downregulation. Heat map and cluster analysis in ALL patients and control cases (FDR *P* value <0.05) were given in Figure 1. Confirmation analysis of significantly dysregulated miRNAs in the patient group by real time RT-PCR revealed 5 upregulated miRNAs (miR-128, miR-146a, miR-155, miR-181a, and miR-195) in ALL (Table 3). Following 6 months of treatment, the level of miR-146a, miR-155, miR-181a, and miR-195 which were found to be upregulated at diagnosis and validated by real time RT-PCR decreased significantly (Table 3). The change in expression level of miR-128, though decreased by 50%, was not significant throughout this period. The expression change of these relevant miRNAs was also presented in Figure 2.

The most significantly upregulated miRNAs and their fold changes in T-lineage ALL and B-lineage ALL are summarized in supplemental Table 1 (see Supplementary Table  1 in Supplementary Material available online at http://dx.doi.org/10.1155/2014/967585). miR-548i, miR-3140, and miR-181b in T-lineage ALL and miR-708, miR-181b, and miR-369-3p in B-lineage ALL were the most distinctive miRNAs among all.

## 4. Discussion

In this study a total of 1136 miRNAs were studied in children with ALL using microarray platform to reveal their contribution with regard to diagnosis, classification, and treatment period. Microarray study revealed 15 miRNAs dysregulated, when compared to the control cases. The results of real time RT-PCR for 5 miRNAs (miR-128, miR-146a, miR-155, miR-181a, and miR-195) were consistent with our microarray results; however, the other 10 miRNAs (miR-548i, miR-708, miR-181b, miR-369-3p, miR-449a, miR-3121, miR-181a, miR-1323, miR-587, and miR-181a-2) contradicted the results of our microarray study. This discrepancy may be due to the small sample size (*n* = 43) used for microarray assay [16]. Array platforms are specifically used for high throughput studies to define significantly dysregulated miRNAs and involve background correction, normalization, and summarization, from which it is recommended that the results be confirmed via other methods. Real time RT-PCR is accepted as a “gold standard” for the confirmation of array platforms [17]; therefore, our data is based on RT-PCR results. After 6 months of follow-up period following chemotherapy, miRNA expression profiles were reevaluated, and their potential involvement in cancer biology was assessed. As presented in Table 3 and shown in Figure 2, the level of all oncogenic miRNAs except miR-128 which were confirmed by real time RT-PCR significantly decreased. This data has not been reported in the literature.

*miR-155* is an oncogenic miRNA which has been shown to be dysregulated in many studies and is suggested as a putative prognostic factor [18, 19]. After 6 months of followup including chemotherapy, it was found to be downregulated in our study. It can be speculated that this result is the reflection of the fact that after 6 months, blasts which overexpress miR-155 have disappeared. Although miR-155 is a potent inhibitor of myeloid differentiation and was found to be upregulated particularly in AML patients [20, 21], it has been reported that this miRNA is also an important factor in lymphopoiesis and immune response [22–24]. The study by Wang et al. has shown that miR-155 was expressed at a significantly higher level in ALL than in AML [25]; therefore, it can be speculated that, in view of our data combined with the literature, miR-155 could be used as a valuable biomarker in the diagnosis and maintenance of ALL.

*miR-146* has been reported to have a role in innate immunity by downregulating* TRAF6* and* IRAK1* genes, and miR-146a is regulated by* NF-kappa* [26, 27]. Combined with miR-155 and miR-181a, which were found to be overexpressed in our study, those 3 miRNAs are associated with genes involved in innate immunity and inflammation, including* TLR4*,* TLR8*,* IRF8*, and* IL6R* [28]. In the light of the information reported in the literature, altered expression of miR-146a, miR-155, and miR-181a could be suggested as additional factors which may lead to leukemia development by causing deleterious effect on normal immune function [29, 30].* miR-181a* has been reported to have a role in the development and in the differentiation of both B cells and cytotoxic T cells [31, 32]. It has also been shown that miR-181a repressed the expression of genes which play a role in thymocyte maturation, such as* Bcl-2*,* CD69*, and the T-cell receptor, and the members of the miR-181 family were found to be significantly overexpressed in T-cell leukemia in our study. These findings support the suggestion that future studies should focus on miR-181 family in the management of ALL.

Studies have shown that* miR-195* has a regulatory function in the cell cycle and cell proliferation [33, 34]. It is expressed in many cancers including chronic lymphocytic leukemia which was one of the 5 most expressed in our patients [35]. However, there is no study in the literature regarding the impact of this miRNA on acute leukemia patients.

*miR-128* together with let-7b and miR-223 was found to be the most discriminatory miRNAs involving ALL and AML [36, 37]. miR-128 was found to be expressed at a significantly higher level in ALL than in AML; therefore, in the light of our results, miR-128 can be used to diagnose and discriminate ALL and AML cases accurately.

Some miRNAs are already known to be related to specific subtypes of pediatric ALL. As reported by Schotte et al., genetic subtypes such as MLL-rearranged, TEL-AML1 positive, hyperdiploid, and drug-resistant leukemic cells display characteristic miRNA signatures in pediatric ALL [38]. Fulci et al. have studied 470 miRNAs in adult ALL patients and identified miRNAs discriminative of the ALL subsets, namely, T-cell and B-cell ALL [39]. miRNAs which were highly discriminative of the different subgroups in this study were not consistent with the results of the present study. The inconsistency between these two studies may be caused by the different age group characteristics. Wang et al. studied 23 miRNAs, which were reported to be associated with hematopoiesis and/or leukemogenesis, to show differentially expressed miRNAs in ALL and AML [25]. Among those identified as differentially expressed miRNAs, miR-128 is remarkable because it also showed significant overexpression in our study.

The limitation of our study lies in the studying of expression levels in specimens containing both normal and blast cells where the results may be affected by the contribution of normal cells. The mean blast cell level in our study, however, was found to be 85% (range: 52–100) meaning this issue may be avoided.

When the age group and its characteristics of childhood leukemia are considered, our data could add important contributions to the literature. The first is the studying of the largest miRNA profile in ALL and the presentation of novel miRNAs associated with leukemogenesis; the second contribution is identifying miRNAs as discriminative of T-lineage versus B-lineage ALL. Moreover, our results confirmed the importance of certain miRNAs such as miR-128, miR-146a, and miR-181a in childhood ALL. The final and possibly the most important contribution is the prospective design of our study that we were able to evaluate miRNAs throughout a treatment period.

In conclusion, the discovery of miRNAs and their association with disease have provided valuable information on potential diagnostic and/or prognostic biomarkers, as well as monitoring the disease progression. In our study, miR-128, miR-146a, miR-155, miR-181a, and miR-195 were found to be significantly dysregulated which may help provide new insights into the diagnosis and prognosis of childhood ALL. Further studies, with larger subject numbers, are needed to clearly demonstrate the effect of miRNAs in leukemogenesis and its practical implications.

## Supplementary Material

“Significant miRNA profile compared to control cases by microarray study in T-lineage ALL and B-lineage ALL and their fold changes are given. Upregulated miRNAs are shown in bold. No significant miRNA was detected after FDR correction in died ALL patients”

## Figures and Tables

**Table 1:** Demographics, laboratory results, and response to the treatment in ALL patients.

Sex	Female *n* (%)	21 (48.8)
	Male *n* (%)	22 (51.2)
Mean age (year old)		6.8 ± 4.5
White blood cells	Mean (mm^3^)	47.930
	Range (mm^3^)	487–474.000
	Subtype	T-lineage: 9
		B-lineage: 34
Blast BM	Mean (%)	85
	Range (%)	52–100
Blast PB	Mean (%)	51
	Range (%)	2–100

Characteristic	Status	*n* (%)

Risk group	Standard risk	13 (30)
	Intermediate risk	20 (47)
	High risk	10 (23)
Steroid response	Good	36
	Poor	7
Response at 33 day	Remission	43
	Not remission	0
Survival	Alive	39
	Dead	4
Relapse	Yes	0
	No	39
	Dead	4

ALL: acute lymphoblastic leukemia, BM: bone marrow, PB: peripheral blood.

**Table 2:** Significant miRNA profile compared to control cases in the microarray study and validation results in real time RT-PCR. According to mean values, only those miRNAs whose “fold change” value demonstrated ± 2-fold or more expression difference were included in the study. miRNAs whose “False Discovery Rate” (FDR) corrected *P* value <0.05 were considered significant. FDR is calculated by using CLC Main Workbench 5 (CLC Bio, Denmark). A total of 13 miRNAs showed upregulation (shown in bold) and 2 miRNAs showed downregulation.

miRNA	Microarray					High/Low Expression (*n*)*	RT-PCR				High/Low Expression (*n*)*
	Control	ALL	Ratio	*P* value	FDR *P*		Control	ALL	log_2_⁡RR	*P*	
**hsa-miR-548i**	1.84	22.12	12.50	3.90*E* − 03	0.03	23/0	0.000112	0.007942	6.15	0.590	21/0
**hsa-miR-708**	46.55	488.77	10.00	6.55*E* − 04	0.01	21/7	0.002949	19.01167	12.65	0.291	28/3
**hsa-miR-181b**	72.46	467.47	6.25	7.09*E* − 06	3.83*E* − 04	36/3	0.0468	48.42431	10.02	0.251	33/2
**hsa-miR-449a**	6.66	23.84	3.57	2.69*E* − 03	0.03	14/0	0.003653	0.013143	1.85	0.163	7/0
**hsa-miR-146a**	326.65	1135.73	3.45	8.31*E* − 05	1.79*E* − 03	35/2	0.068189	24.37251	8.48	0.008	35/1
**hsa-miR-155**	170.68	515.25	3.03	7.93*E* − 05	1.79*E* − 03	32/2	0.063027	31.66987	8.97	0.009	34/0
hsa-miR-181a*	104.93	298.17	2.86	6.54*E* − 03	0.04	25/4	0.012341	0.766604	5.96	0.123	35/2
**hsa-miR-3121**	12.04	33.64	2.78	6.95*E* − 03	0.04	21/0	0.023803	0.001278	−4.22	0.209	0/0
**hsa-miR-181a**	661.35	1810.68	2.70	3.66*E* − 03	0.03	29/7	0.770556	80.63069	6.71	0.002	34/2
**hsa-miR-128**	720.72	1713.49	2.38	2.67*E* − 06	2.88*E* − 04	30/0	0.245556	11.3962	5.54	0.009	33/4
**hsa-miR-1323**	34.21	81.96	2.38	1.05*E* − 03	0.01	25/2	0.000686	0.000024	−4.84	0.199	0/0
**hsa-miR-195**	962.76	2251.43	2.33	9.67*E* − 04	0.01	28/4	0.012433	1.250504	6.65	<0.001	32/3
**hsa-miR-587**	33.83	69.93	2.08	5.67*E* − 03	0.04	17/4	0.000523	0.000146	−1.84	0.326	1/0
hsa-miR-640	91.53	45.07	−2.03	7.18*E* − 03	0.04	3/30	0.050227	0.000498	−6.66	0.191	0/0
hsa-miR-145	498.27	197.73	−2.52	8.09*E* − 05	1.79*E* − 03	2/35	1.201444	14.55961	3.60	0.440	26/10

FDR: false discovery rate, ALL: acute lymphoblastic leukemia.

*“High/Low Expression (*n*)” means the number of cases in which particular miRNA expression is over the normal range. Normal range was accepted for the 95% CI of control cases.

**Table 3:** Significant miRNAs after validation by real time RT-PCR at day 0 were given. Those miRNAs were reevaluated after 6 months of treatment and the expression change of all miRNAs except miR-128 was significant.

	miRNA	RR
Validated by RT-PCR (at diagnosis)	hsa-miR-128	11.396200
	hsa-miR-146a	24.372519
	hsa-miR-155	31.669875
	hsa-miR-181a	80.630694
	hsa-miR-195	1.250504

Real time RT-PCR (after 6 months)	hsa-miR-128	6.073660
	hsa-miR-146a	0.334798
	hsa-miR-155	1.201844
	hsa-miR-181a	4.987600
	hsa-miR-195	0.206393

RR: relative ratio.
