# Supplementary material for: Prospective Evaluation of Whole Genome MicroRNA Expression Profiling in Childhood Acute Lymphoblastic Leukemia
Source: Biomed Res Int. 2014 May 14;2014:967585. doi: 10.1155/2014/967585 (PMC4053274; doi:10.1155/2014/967585)
Supplement: Supplementary file 1 — “Significant miRNA profile compared to control cases by microarray study in T-lineage ALL and B-lineage ALL and their fold changes are given. Upregulated miRNAs are shown in bold. No significant miRNA was detected after FDR correction in died ALL patients” [file 967585.f1.pdf]

Supplemental Table 1. Significant miRNA profile compared to control cases by microarray study in T-lineage ALL and B-lineage ALL. No significant miRNA was detected after FDR correction in died ALL patients (upregulated miRNAs are shown in bold)

T-lineage ALL		B-lineage ALL		Dead (B-lineage)	
**miRNA**	**Fold Change**	**miRNA**	**Fold Change**	**miRNA**	**Fold Change**
**hsa-miR-548i**	20,00	**hsa-miR-708**	12,50	**hsa-miR-548i**	25.0
**hsa-miR-3140**	14,29	**hsa-miR-181b**	5,26	hsa-miR-3191	-2.43
**hsa-miR-181b**	11,11	**hsa-miR-369-3p**	4,00	hsa-miR-4296	-2.78
**hsa-miR-3115**	11,11	**hsa-miR-146a**	3,57	hsa-miR-513a-5p	-3.2
**hsa-miR-548d-5p**	8,33	**hsa-miR-155**	2,94		
**hsa-miR-449a**	6,25	**hsa-miR-195**	2,70		
**hsa-miR-181c**	6,25	**hsa-miR-128**	2,44		
**hsa-miR-181a**	5,56	hsa-miR-143	-2,24		
**hsa-miR-181a***	5,56	hsa-miR-145	-2,50		
**hsa-let-7b***	5,00				
**hsa-miR-1827**	4,55				
**hsa-miR-92a-1***	3,85				
**hsa-miR-299-3p**	3,85				
**hsa-miR-155**	3,33				
**hsa-miR-181a-2***	3,23				
**hsa-miR-1323**	2,94				
**hsa-miR-587**	2,70				
**hsa-miR-7-1***	2,63				
**hsa-miR-28-3p**	2,38				
**hsa-miR-130b**	2,33				
**hsa-miR-27b**	2,22				
**hsa-miR-361-5p**	2,08				
hsa-miR-633	-2,04				
hsa-miR-326	-2,08				
hsa-miR-501-3p	-2,11				
hsa-miR-802	-2,15				
hsa-miR-4260	-2,17				
hsa-miR-3130-5p	-2,26				
hsa-miR-145	-2,59				
hsa-miR-186	-2,68				
hsa-miR-593*	-2,76				
hsa-miR-574-3p	-2,79				
hsa-miR-4262	-2,81				
hsa-miR-640	-3,03				
hsa-miR-606	-3,62				

ALL: Acute lymphoblastic leukemia
